# Research of N-acetyl-L-cysteine on CD40–CD40L pathway in pulmonary fibrosis induced by silicon dioxide

**DOI:** 10.3389/fgene.2026.1845040

**Published:** 2026-06-18

**Authors:** Shujuan Wang, Lili Wang, Kui Hu, Jingyin Han, Xiaoyu Gan, Yi Lou, Guohui Li

**Affiliations:** Department of Occupational Medicine, Hangzhou Red Cross Hospital, Hangzhou, Zhejiang, China

**Keywords:** CD40–CD40L, collagen I, N-acetyl-L-cysteine, silicosis, α-smooth muscle actin (α-SMA)

## Abstract

**Introduction:**

To investigate the effect of N-acetyl-L-cysteine (NAC) on the CD40–CD40L signaling axis during the development of pulmonary fibrosis in silicosis.

**Methods:**

Seventy-five patients treated in our department between January 2018 and June 2023 were enrolled and allocated to five groups (n = 15 each): healthy control, silicosis, routine treatment, intervention-1, and intervention-2. Bronchoalveolar lavage fluid (BALF) was collected from all subjects. CD40L expression on T lymphocyte surfaces was measured by flow cytometry. BALF concentrations of interleukin-6 (IL-6), interleukin-8 (IL-8), interferon-γ (IFN-γ), and monocyte chemoattractant protein-1 (MCP-1) were quantified by ELISA. BALF samples from each group were co-cultured with the human fetal lung fibroblast cell line HFL-1; Collagen I expression was assessed by immunocytochemistry and α-smooth muscle actin (α-SMA) expression by Western blotting.

**Results:**

T cells isolated from BALF of silicosis patients exhibited significantly higher surface CD40L expression than those from healthy controls. Both intervention groups showed marked reductions in T-cell CD40L expression compared with the untreated silicosis group, with the greatest decrease in intervention-2. BALF levels of IL-6, IL-8, IFN-γ, and MCP-1 were significantly elevated in silicosis patients versus controls. Compared with the silicosis group, the routine treatment, intervention-1, and intervention-2 groups demonstrated progressive reductions in these cytokines, most pronounced in intervention-2. HFL-1 cells cultured with silicosis BALF displayed significantly increased α-SMA and Collagen I expression relative to cells exposed to control BALF. BALF from the routine treatment group produced no significant change in HFL-1 α-SMA fluorescence versus the silicosis group, whereas BALF from intervention-1 and intervention-2 significantly reduced HFL-1 α-SMA and Collagen I expression, with intervention-2 yielding the largest effect.

**Discussion:**

NAC downregulates T-cell surface CD40L expression and disrupts CD40–CD40L interactions, attenuating production of multiple proinflammatory cytokines, reducing collagen synthesis and deposition, and thereby delaying the progression of interstitial pulmonary fibrosis in silicosis.

## 1 Introduction

Occupational silicosis is a severe and irreversible occupational lung disease that poses a serious threat to workers’ health. In recent years, with the continuous advancement of research, investigations into silicosis-associated pulmonary fibrosis have extended from histopathological observations to the cellular and molecular levels, including studies on effector cells, cytokines, and signal transduction pathways (Cavalin, 2019; He et al., 2024). Modulation of effector cells and cytokines involved in pulmonary fibrosis, particularly targeting key signaling molecules that mediate fibrotic processes, may provide novel therapeutic strategies for the prevention and treatment of pulmonary fibrosis. Previous studies, together with our preliminary experimental findings, have demonstrated that the CD40–CD40L interaction system, as an important immune-inflammatory signaling pathway, may play a critical role in the development of silicosis, especially during the acute alveolar exudative phase and the subsequent fibrotic stage (Krefft et al., 2020; Wang and Han, 2015). Therefore, targeting this signaling pathway and suppressing excessive inflammatory responses may represent a promising approach for the treatment of silicosis-induced pulmonary fibrosis. N-acetyl-L-cysteine (NAC), a well-known sulfhydryl (–SH) donor, exhibits potent antioxidant, anti-inflammatory, and anti-fibrotic properties (Santus et al., 2024). Accumulating evidence indicates that high-dose NAC can modulate multiple signaling pathways and downregulate the expression of pro-fibrotic mediators, such as transforming growth factor-β (TGF-β), tumor necrosis factor-α (TNF-α), and various interleukins, thereby inhibiting fibroblast proliferation and collagen synthesis and ultimately attenuating the progression of pulmonary interstitial fibrosis (Saha and Talwar, 2024). As an anti-fibrotic agent, NAC has shown therapeutic potential in idiopathic pulmonary fibrosis (IPF) and chronic obstructive pulmonary disease (COPD) (Mokra et al., 2023). However, studies focusing on the role of NAC in silicosis-associated pulmonary fibrosis remain limited, and its regulatory effects on the CD40–CD40L signaling pathway have not been fully elucidated. Therefore, the present study aims to investigate the effects of NAC on the CD40–CD40L interaction system and to further explore whether NAC can reduce the release of inflammatory cytokines, inhibit collagen synthesis, and ultimately prevent the development of pulmonary fibrosis. These findings may provide experimental evidence and a theoretical basis for identifying effective therapeutic strategies against silicosis-induced pulmonary fibrosis.

## 2 Methods

### 2.1 Clinical data

A total of 75 subjects were enrolled from the outpatient and inpatient departments of our hospital between January 2018 and June 2023. All participants were male, aged between 50 and 80 years. The subjects were divided into five groups (n = 15 per group): healthy control group, silicosis group, regular treatment group, intervention treatment-1 group, and intervention treatment-2 group.

Control group: Subjects who underwent fiberoptic bronchoscopy due to chronic cough, foreign body sensation in the throat, or similar symptoms, but showed no obvious abnormalities on bronchoscopic examination or clinical diagnosis.

Silicosis group: Patients diagnosed with silicosis, including 8 cases of stage I, 4 cases of stage II, and 3 cases of stage III pneumoconiosis.

Regular treatment group: Patients with silicosis (7 cases of stage I, 5 cases of stage II, and 3 cases of stage III) who received conventional anti-silicosis and anti-fibrotic treatment.

Intervention treatment-1 group: Patients with silicosis (8 cases of stage I, 5 cases of stage II, and 2 cases of stage III) who received conventional treatment combined with low-dose N-acetylcysteine (NAC) at 600 mg once daily for 3 months.

Intervention treatment-2 group: Patients with silicosis (6 cases of stage I, 6 cases of stage II, and 3 cases of stage III) who received high-dose NAC at 600 mg three times daily for 3 months.

The diagnosis and staging of pneumoconiosis were based on the national standard GBZ 70–2015. This study was approved by the Ethics Committee of the hospital, and written informed consent was obtained from all participants.

### 2.2 Inclusion and exclusion criteria

Inclusion criteria

1. Diagnosis consistent with the above diagnostic criteria;
2. Male patients aged 50–80 years;
3. Provision of written informed consent.

Exclusion criteria

1. Patients with extremely severe pulmonary dysfunction;
2. Patients with severe comorbid diseases involving the cardiovascular, hepatic, renal, or other major organ systems.

### 2.3 Laboratory materials

1. The human fetal lung fibroblast cell line HFL-1 was purchased from Shanghai Tongpai Biotechnology Co., Ltd. Cells were routinely cultured in high-glucose DMEM supplemented with 10% fetal bovine serum (FBS) and passaged every 3 days. Cells in the logarithmic growth phase between passages 16 and 25 were used for experiments.
2. Bronchoalveolar lavage fluid (BALF) collected from the clinical study was added to the culture medium for stimulation and co-culture of HFL-1 cells.

### 2.4 Experimental methods

#### 2.4.1 *In vivo* experiments

##### 2.4.1.1 Collection of bronchoalveolar lavage fluid (BALF)

BALF samples were obtained from the affected lung middle lobe or lingular segment using fiberoptic bronchoscopy performed by experienced bronchoscopists in strict accordance with the Guidelines for Clinical Application of Fiberoptic Bronchoscopy. Briefly, 100–150 mL of sterile 0.9% saline warmed to 37 °C was instilled into the bronchoalveolar space in three aliquots through a lavage catheter and gently aspirated using negative pressure. A recovery rate greater than 40% was considered successful. BALF samples were filtered through double-layer gauze, cooled at 4 °C for 10 min, and processed for subsequent experiments.

Adherent cells (monocytes and macrophages) were removed using the adherence method, and BALF mononuclear cells were isolated and purified by density gradient centrifugation.

##### 2.4.1.2 Flow cytometric analysis of CD40L expression on T lymphocytes

BALF mononuclear cells were adjusted to a concentration of 1 × 10^5^ cells/mL and subjected to direct immunofluorescence staining. Cells were incubated with PE-conjugated mouse anti-human CD40L monoclonal antibody, with PE-labeled mouse IgG used as a negative control. After incubation at 4 °C in the dark for 20 min, cells were centrifuged, resuspended in 100 μL PBS, washed three times, and fixed with 1% paraformaldehyde.

T lymphocytes were analyzed using a FACStar flow cytometer (Becton Dickinson, Mountain View, CA). Mean fluorescence intensity (MFI) was used to quantify CD40L expression.

##### 2.4.1.3 Measurement of cytokine levels in BALF

The concentrations of interleukin-8 (IL-8), interleukin-6 (IL-6), interferon-γ (IFN-γ), and monocyte chemoattractant protein-1 (MCP-1) in BALF were determined using enzyme-linked immunosorbent assay (ELISA) kits according to the manufacturers’ instructions. Absorbance was measured at 450 nm using a microplate reader, and cytokine concentrations were calculated based on standard curves.

#### 2.4.2 *In vitro* experiments

##### 2.4.2.1 HFL-1 cell stimulation

BALF prepared as described above was added to the culture medium to stimulate HFL-1 fibroblasts for subsequent analyses.

##### 2.4.2.2 Immunocytochemistry and protein expression analysis

Immunocytochemical staining was performed using a monoclonal antibody against collagen type I at a dilution of 1:100. Cells were incubated overnight at 4 °C in a humidified chamber. PBS was used instead of the primary antibody as a negative control. The remaining steps were performed according to the kit instructions.

Protein concentrations in culture supernatants were determined using ultraviolet spectrophotometry. Equal amounts of total protein were mixed with loading buffer and subjected to SDS-PAGE, followed by transfer to nitrocellulose membranes for Western blot analysis. The primary antibody was diluted 1:500, and collagen type I standard protein and PBS were used as controls.

Stained cell slides were analyzed using the Beihang automatic image analysis system. Six random fields were selected per slide, and the mean integrated optical density (IOD) was used as a quantitative indicator. Western blot bands were quantified using Quantity One software.

##### 2.4.2.3 Total protein extraction

HFL-1 cells were seeded into 100 mL culture flasks. Upon reaching approximately 80% confluence, cells were serum-starved in medium containing 0.5% FBS for 24 h. Cells were lysed with 40–60 μL lysis buffer per flask on ice for 30 min, followed by ultrasonication for 30 s. Lysates were centrifuged at 12,000 rpm for 8 min, and the supernatants were collected and stored at −20 °C. Protein concentrations were determined using a bicinchoninic acid (BCA) assay kit (Pierce, United States).

##### 2.4.2.4 Western blot analysis

Total protein (100 μg) was mixed with an equal volume of 2× SDS loading buffer, boiled for 3 min, and separated by 10% SDS–polyacrylamide gel electrophoresis (PAGE). Proteins were transferred to nitrocellulose membranes at a constant voltage of 15 V for 45 min. Membranes were stained with Ponceau S to confirm equal loading, blocked with 5% non-fat milk for 1 h, and incubated with mouse monoclonal anti-α-SMA antibody (1:100) at room temperature for 8–12 h.

After washing with PBS, membranes were incubated with rabbit anti-mouse secondary antibody (1:1,000) for 2 h, followed by DAB color development. Bands were scanned and analyzed using Image Tool 3.0, with Ponceau S staining used for normalization.

### 2.5 Statistical analysis

Statistical analysis was performed using SPSS version 16.0 (SPSS Inc., Chicago, IL, United States). Data are expressed as mean ± standard deviation (SD). Flow cytometry data were analyzed using FACStar software, and CD40L expression was evaluated by mean fluorescence intensity (MFI). Immunofluorescence images were analyzed using ImageJ software to determine the average optical density (AOD) of α-SMA expression.

Comparisons between two groups were performed using Student’s *t*-test for continuous variables and the χ^2^ test for categorical variables. A *P* value <0.05 was considered statistically significant, and *P* < 0.01 was considered highly statistically significant.

## 3 Results

### 3.1 Baseline comparability between groups

Baseline characteristics, including sex and age distribution, were compared between the groups to assess comparability. The results showed no significant differences in sex or age between the groups (*P* > 0.05), indicating good baseline balance.

During the study period, one patient in the regular treatment group withdrew due to the development of pneumothorax, and one patient in the intervention treatment-1 group withdrew because of gastric ulcer, which prevented continued oral medication.

### 3.2 Levels of inflammatory cytokines in BALF

**FIGURE 1:**
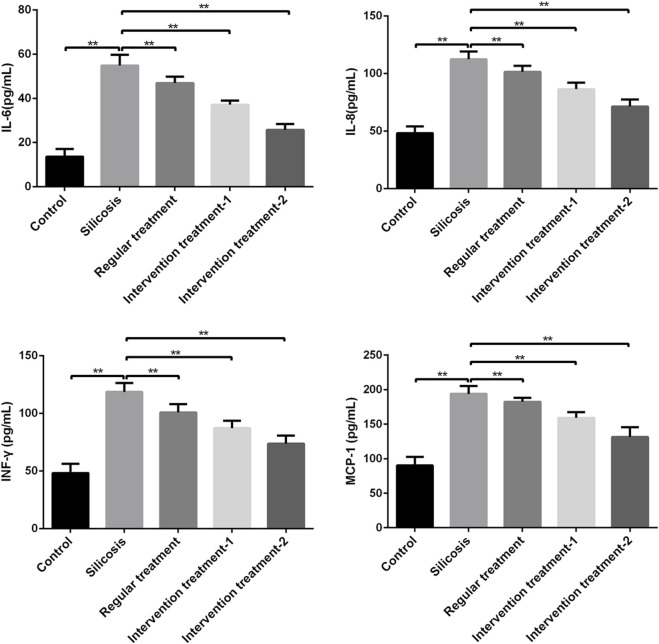
Concentrations of IL-6, IL-8, INF-γ and MCP-1 in BALF samples of each group.

Figure 1 presents the ELISA results of bronchoalveolar lavage fluid (BALF) samples from each group. Compared with the healthy control group, the levels of interleukin-8 (IL-8), interleukin-6 (IL-6), interferon-γ (IFN-γ), and monocyte chemoattractant protein-1 (MCP-1) were significantly elevated in the BALF of patients with silicosis (*P* < 0.05 or *P* < 0.01).

Compared with the silicosis group, the concentrations of IL-8, IL-6, IFN-γ, and MCP-1 were reduced to varying degrees in the regular treatment group, intervention treatment-1 group, and intervention treatment-2 group. Among these, the intervention treatment-2 group exhibited the most pronounced reductions in inflammatory cytokine levels.

### 3.3 Expression of CD40L on T lymphocytes

**FIGURE 2:**
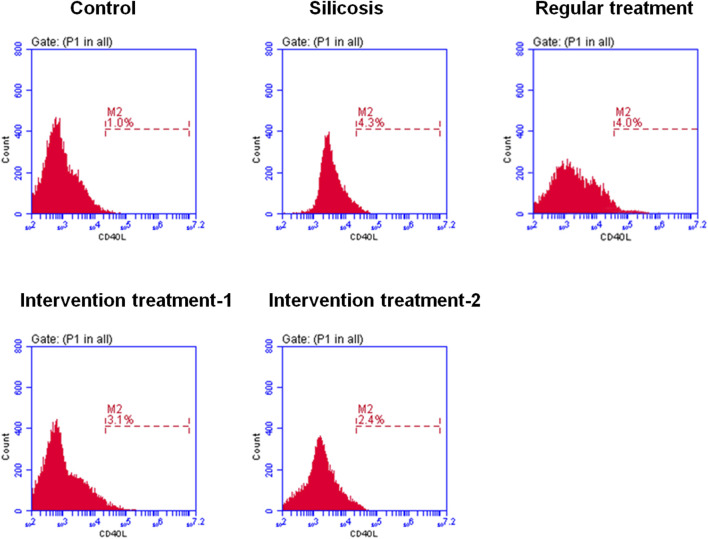
Flow cytometry detection results of CD40L on the surface of T lymphocytes in each group.

Figure 2 shows representative flow cytometric plots and quantitative analysis of CD40L expression on the surface of T lymphocytes. Compared with T lymphocytes isolated from the BALF of the healthy control group, CD40L expression on T lymphocytes isolated from the BALF of patients with silicosis was significantly increased (*P* < 0.05).

Compared with the silicosis group, no significant difference in CD40L expression was observed in the regular treatment group (*P* > 0.05). In contrast, CD40L expression on T lymphocytes was significantly decreased in both the intervention treatment-1 and intervention treatment-2 groups (*P* < 0.05 or *P* < 0.01), with a more marked reduction observed in the intervention treatment-2 group.

### 3.4 Collagen I expression in HFL-1 cells

**FIGURE 3:**
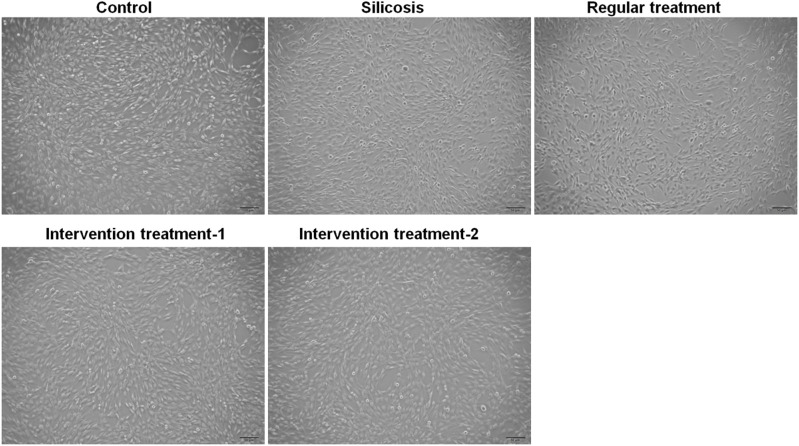
HFL-1 cells cultured from bronchoalveolar lavage fluid of each group.

**FIGURE 4:**
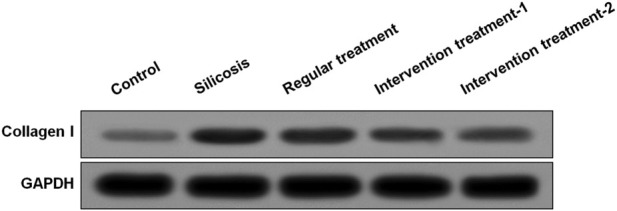
Collagen I protein expression in cells of each group.

**FIGURE 5:**
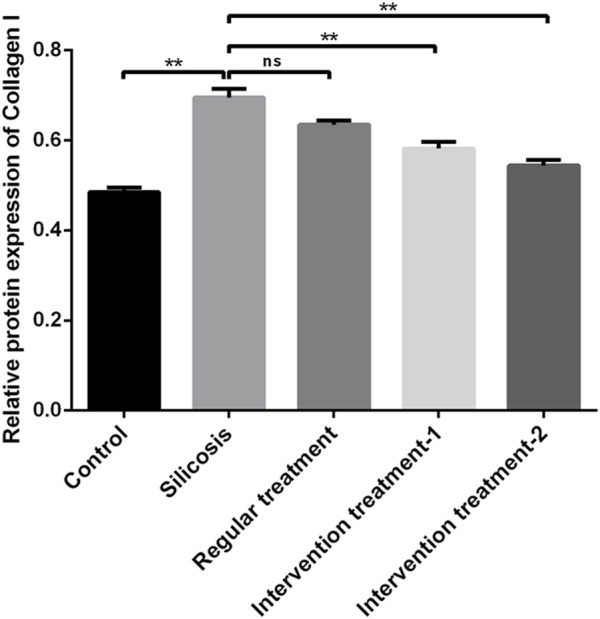
Statistical analysis of Collagen I protein expression in cells from each group.

Figure 3 illustrates the morphological changes of HFL-1 cells after stimulation with BALF from different groups, while Figures 4, 5 show the expression levels of collagen type I protein.

Compared with HFL-1 cells cultured with BALF from the healthy control group, exposure to BALF from patients with silicosis resulted in a significant increase in collagen I protein expression (*P* < 0.05). Compared with the silicosis group, no significant difference in collagen I expression was observed in HFL-1 cells treated with BALF from the regular treatment group (*P* > 0.05).

In contrast, HFL-1 cells treated with BALF from the intervention treatment-1 and intervention treatment-2 groups exhibited significantly reduced collagen I expression compared with the silicosis group (*P* < 0.05 or *P* < 0.01), with the greatest reduction observed in the intervention treatment-2 group.

### 3.5 α-SMA expression in HFL-1 cells

**FIGURE 6:**
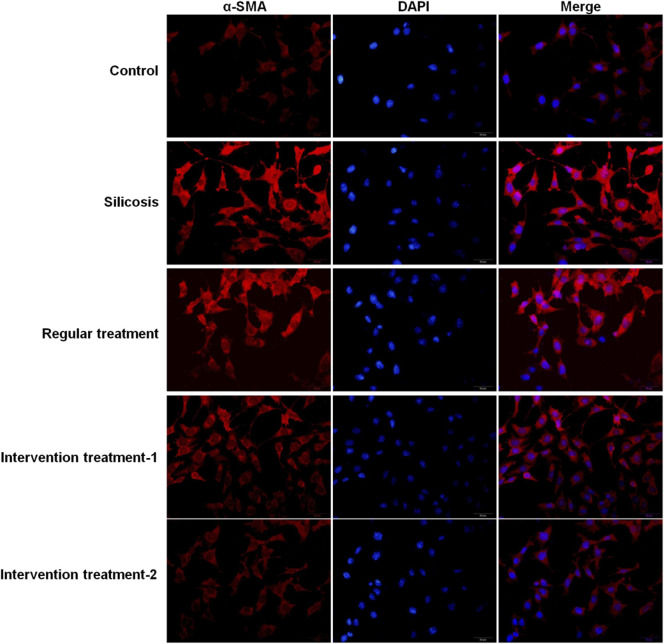
Immunofluorescence images of α-SMA in HFL-1 cells cultured with bronchoalveolar lavage fluid from each group.

Figure 6 shows representative immunofluorescence images of α-smooth muscle actin (α-SMA) expression in HFL-1 cells following treatment with BALF from different groups.

Compared with HFL-1 cells cultured with BALF from the healthy control group, α-SMA fluorescence intensity was significantly increased in cells treated with BALF from patients with silicosis (*P* < 0.05). Compared with the silicosis group, no significant difference in α-SMA expression was observed in cells treated with BALF from the regular treatment group (*P* > 0.05).

However, treatment with BALF from the intervention treatment-1 and intervention treatment-2 groups significantly attenuated α-SMA expression in HFL-1 cells compared with the silicosis group (*P* < 0.05 or *P* < 0.01), with the most pronounced reduction observed in the intervention treatment-2 group.

## 4 Discussion

Occupational diseases are associated with high rates of disability and mortality, often resulting in the loss of the primary workforce within affected families and leading to severe social consequences, including disease-related disability, poverty, and family disruption (Barnes and Glaspole, 2023). Silicosis is a representative occupational lung disease characterized by continuous disease progression even after cessation of dust exposure. Patients are prone to complications such as respiratory infections and cor pulmonale, which impose a substantial economic burden on healthcare systems and society as a whole (Peruzzi et al., 2022). However, the pathogenic mechanisms underlying silicosis-associated pulmonary fibrosis have not been fully elucidated, and effective therapeutic options remain limited.

Recent studies have demonstrated that the CD40–CD40L interaction system serves as an essential costimulatory signaling pathway for immune activation and plays a critical role in the development of various inflammatory and immune-mediated diseases (Tariket et al., 2019). Based on our previous investigations, we hypothesized and subsequently confirmed that the CD40–CD40L system mediates immune-inflammatory responses primarily through interactions between T lymphocytes and macrophages acting as antigen-presenting cells (APCs). In this process, major histocompatibility complex (MHC) molecules on macrophages provide the primary activation signal to T cells, inducing CD40L expression. The binding of CD40L on T cells to CD40 on macrophages then delivers a secondary costimulatory signal, promoting T-cell activation and interleukin-12 (IL-12) secretion. This cascade results in an imbalance of the Th1/Th2 immune response and excessive release of pro-inflammatory and pro-fibrotic cytokines, including interleukin-8 (IL-8), interleukin-6 (IL-6), interferon-γ (IFN-γ), and monocyte chemoattractant protein-1 (MCP-1) (Gardner et al., 2018; Wang et al., 2020). These mediators further stimulate fibroblast biosynthetic activity, ultimately leading to fibroblast proliferation and extracellular matrix accumulation. Accordingly, the CD40–CD40L pathway is considered a key activator of fibroblasts and other structural cells involved in pulmonary fibrosis.

In the present study, CD40L expression on T lymphocytes isolated from bronchoalveolar lavage fluid (BALF) of patients with silicosis was significantly increased compared with that of healthy controls, which is consistent with previous findings. Importantly, after treatment with different doses of N-acetylcysteine (NAC), CD40L expression on T lymphocytes was significantly reduced in both intervention treatment groups compared with the silicosis group, with a more pronounced reduction observed in the high-dose NAC group. These results indicate that NAC effectively downregulates CD40L expression on T lymphocytes in patients with silicosis.

Previous studies have reported that NAC, a thiol-containing reducing agent, can attenuate oxidative stress, decrease inflammatory cytokine production, inhibit airway epithelial thickening and remodeling, and thereby alleviate pulmonary fibrosis (Zhang et al., 2023; Askari et al., 2020). Consistent with these observations, our results showed that BALF levels of IL-8, IL-6, IFN-γ, and MCP-1 were significantly reduced in both NAC-treated groups compared with the silicosis group, with the greatest reduction observed in the high-dose NAC group. These findings suggest that high-dose NAC may suppress reactive oxygen species generation, modulate cellular redox balance, and inhibit excessive inflammatory cytokine release, thereby attenuating pulmonary inflammatory responses.

Furthermore, *in vitro* experiments demonstrated that BALF from NAC-treated patients significantly reduced the expression of α-smooth muscle actin (α-SMA) and collagen type I in HFL-1 fibroblasts compared with BALF from untreated silicosis patients. Notably, the inhibitory effects were most pronounced in the high-dose NAC group, further confirming that NAC can effectively suppress fibroblast activation and extracellular matrix synthesis, thereby impeding the progression of pulmonary interstitial fibrosis.

## 5 Conclusion

In conclusion, the present study provides additional evidence that the CD40–CD40L interaction system represents a critical inflammatory signaling pathway in the pathogenesis of silicosis. NAC, as a thiol-containing compound and a precursor of glutathione, can downregulate CD40L expression on T lymphocytes, disrupt CD40–CD40L-mediated inflammatory signaling, and suppress the synthesis and release of multiple inflammatory cytokines. These effects attenuate fibroblast activation, inhibit extracellular matrix production, and reduce collagen deposition, ultimately delaying the development of silicosis-associated pulmonary fibrosis. In addition to its anti-inflammatory and anti-fibrotic properties, NAC also exhibits mucolytic activity and reduces bacterial colonization, highlighting its potential clinical value and warranting further investigation in the management of silicosis.

## Data Availability

The datasets presented in this study can be found in online repositories. The names of the repository/repositories and accession number(s) can be found in the article/supplementary material.
